# The American Institute and Its Laughter

**Published:** 1887-04

**Authors:** P. P. Wells

**Affiliations:** Brooklyn, N. Y.


### HOMŒOPATHIC PHYSICIAN,

A MONTHLY JOURNAL OF MEDICAL SCIENCE.

"If our school ever gives up the strict inductive method of Hahnemann, we are lost, and deserve only to be mentioned as a caricature in the history of medicine."—CONSTANTINE HERING.

Vol. VII. APRIL, 1887. No. 4.

### THE AMERICAN INSTITUTE AND ITS LAUGHTER.

P. P. WELLS, M. D., BROOKLYN, N. Y.

"*It* [practice with high Dynamizations] *has been laughed out of the American Institute."—Ex-President of A. I. H.*

This laughter seems to have been powerful, loud, and long, if judged by the effects it has been said to have produced. We know it has been loud and long, for we have heard it. But it is not so apparent that it has always been as wise as it has been loud. It seems to have been quite powerful at times, if we may accept this testimony of an ex-President of this numerous body. If this be true, which is the sufferer, the dynamizations or the Institute? There is further evidence of its power in the effect it seems to have had on the laughers themselves, and especially in this, that it seems to have extinguished in these all sense of responsibility to law [" bound by no law."—*Its President at St. Louis*], and also by the disgraceful Indianapolis resolution; and still further by the disgust it has produced in so many of its best members, and to the extent that they are no more seen in the assembly of these unwise laughers. They are no more willing to be counted with those who, being ignorant of God's law, repudiate it; so ignorant, also, of the best means it employs for the cure of the sick, they affect to blow these into non-existence by their so great explosions of laughter! And further, the power of this greatest of the latest acts of the Institute is manifest in the banishment from their body of all which is characteristic of Homœopathy except the name, and even this, as judged by their transactions, does not seem to have "*struck skin-deep.*" The violence of the laughter has only left them strengh to try and show how much they know of old physic. The result has been rather to show how little they know of Homœopathy. Indeed, this does not seem to have been at all in their thoughts. Alas for them!

We have said we have heard this laughter. We have heard it but once, and then it was in this way: An earnest member, who had been of a mind to work in the direction and interests of Homœopathy, in the simplicity of his heart brought to the Institute *provings* of substances, as Homœopathy requires all agencies shall be proved before they can be admitted into the armamentarium it employs for the cure of sicknesses. This was the very kind of work the founders of this Institute had in mind when the organization was created to enlarge and perfect the knowledge of our materia medica. Did the Institute receive, with thankfulness, this contribution to their knowledge of a subject, of which they then knew absolutely nothing? No! They laughed! They not only laughed at this working member, but they grossly insulted him; and this was his reward for his honest endeavor to add to their knowledge and their means for healing! They laughed considerably, and just as though they would have this understood as evidence that they *knew* a good deal. They knew, and probably now know, just nothing at all of the matter over which they became so hilarious, and before which they exhibited such bad manners. But, after all, their laughter and their boorishness added nothing to their power as healers, nor to the respect which good men would be glad to entertain for their body. And more, though this may be a disappointment to the members, it added but little to the perfection of their attempted imitation of old physic.

This one element in their history, in which they seem so greatly to excel, is the resort with which they endeavor to conceal their abandonment of the Homœopathy of the founders of their body, *i. e.*, the Homœopathy of Hahnemann, and when laughter is found not quite equal to the needs of the case, they have another resort, which seems to give them great satisfaction. This is to praise Dunham.*

---

* This reminds one of the advice given in these words by the accomplished editor of the *Spectator*, to critics who were conscious of inability to deal successfully with difficult subjects: "It will always be safe to say, if the painter had taken more pains the picture would have been better; and *be sure to praise the works of Pietro Perugino!*" No matter whether you know anything of these, his reputation will make this "*safe!*" So of those who praise Dunham. A knowledge of the law, or the principles which govern its administration, which controlled his practice, have not been found necessary to those who praise him. "Praise Perugino," for it sounds well, and for all the world just as though one *understood* and approved it all.

And he is so worthy of all praise they seem to have regarded his merits as equal to covering all their ignorance. They could *laugh* at the prover of these remedies, but Dunham was wise enough to use them successfully. This was, and is, just the difference between him and those who praise him, because, somehow, they have found it is the proper thing to do. They know nothing, and care nothing, for his law, its corollaries, or the loyalty of spirit with which he administered these, but only that the proper thing to do is to "praise Pietro Perugino!" It is a relief if one may safely hope our colleague of so many and so rare excellencies is but ignorant of the practical character of these adulators, and of that of the praise with which they so freely cover him.

This is how this meritorious prescriber dealt with one of these laughed-at remedies. It was so loudly and badly laughed at that it could have no place on the record of the Institute, nor on the pages of that largest work of our materia medica, which professes to give us all known remedies worthy of our respect. This remedy was not known to the Institute, so they laughed at it. If they are consistent, and laugh at all they do not know, they are not likely to lack for subjects for their merriment for some time. Dunham did not join in this unseemly laughter. He studied the record of the remedy rather, and found in it a likeness to his record of a gastrosis for which he had been giving the nearest simillimum he could find in the old record of our long-used remedies for between three and four months without the slightest benefit. It was after the long trial and many disappointments that he turned to the pathogenetic record of *Lac acidum*, and found it an *exact* simillimum to his case.* He knew both the laughter and the exclusion, but these were no bar to his selection of the remedy. He gave it and neither saw nor heard more of his patient till her mother called in response to a bill sent for his services. Then the absence of the patient was explained, after the mother had given an angry inquiry as to why he "had not given this last remedy before?" and had accused him of "keeping her daugh-

* The Institute was assuredly ignorant of the great and most important principle that each material substance has in it a dynamis of specific individuality, which is capable of liberation and development by proper manipulation, and so of being brought into beneficent relation to practical medicine. This is comparatively a modern discovery, to the confirmation of the truth of which this abused member has so largely contributed. They only saw in his proving the material—*sour milk*—and they laughed! They saw nothing of the curing principle in this which brought Dunham and his patient out of their embarrassment. "*First be sure you're right,*" then *laugh!*

ter along to enlarge his bill." After this outburst she said "her daughter was perfectly well, and had not had the least trouble since taking the last remedy." Now was not Dunham's confidence in the remedy better than the unseemly and untimely laugh of the Institute? Was not their laughter, if judged in the light of this cure, a little premature? Then while they praise Perugino, let them endeavor to imitate his works.

